# Cochrane Review Summary: Asthma education for school staff

**DOI:** 10.1017/S1463423619000860

**Published:** 2019-11-12

**Authors:** Daksha Trivedi

**Affiliations:** Senior Research Fellow, Centre for Research in Public Health and Community Care, University of Hertfordshire, Hatfield, UK

**Keywords:** asthma, education, intervention, programme, schools, staff, teachers

## Review question

- To evaluate the effectiveness and safety of asthma education programmes for school staff
- To explore their key characteristics including teaching content and delivery

## Relevance to primary care and nursing

Personalised asthma action plans have been recommended for use in children, although their uptake remains low. Primary health care professionals including specialist nurses have a vital role in the management of asthma. It is important that parents, children and teachers are educated about managing asthma in school children (SIGN 158, 2019).

## Characteristics of the evidence

This Cochrane review contains five cluster randomised controlled trials involving 111 schools, of which three studies included teachers as participants (*n* = 2008), one included families with a child diagnosed with asthma (*n* = 591), and one included children with asthma (*n* = 209) as participants (Kew *et al*., 2017).

Included studies targeted all types of school staff, from any type of school with pupils aged under 20.

Follow-up ranged from 1 to 12 months. Included studies compared staff asthma education with no education or a minimal intervention control. They provided educational programmes or training sessions aimed to educate school staff about how to manage asthma and respond to an asthma attack. Three studies included pupil workshops and one provided training for the pupils’ primary care providers. Studies involving multiple components other than asthma education were excluded.

Two studies were conducted in the UK, and one in the USA, Jordan, and Australia, respectively. Interventions were delivered by researchers, trained and health care professionals including school nurses, pharmacists, and physicians.

## Summary of key evidence

All studies were assessed as high risk of bias, primarily due to selection bias and allocation concealment issues. Pooled evidence was of low quality overall judged using GRADE (The Grading of Recommendations Assessment, Development and Evaluation), mostly due to inherent biases related to trial design. There was considerable heterogeneity between the studies.

Primary outcomes: visits to emergency department (ED) or hospital, mortality, asthma control, or knowledge.

Secondary outcomes: adverse events, adherence to asthma policies, absenteeism related to asthma staff self-efficacy, and preparedness.

Other qualitative: educational content (primary material, learning outcomes, theoretical underpinning), teaching attributes of training programmes used (staff and resource requirements, length of course, any follow-up service or session).

Continuous data were summarised as mean differences (MD) or standardised mean differences (SMD), if different scales were combined, along with 95% confidence intervals (CI). Dichotomous data were summarised as odds ratio (OR) with 95% CI. The findings are summarised for outcomes where data were available and reported.

*Visits to ED/hospital over previous 12 months*: Only one study reported mean number of visits per child in both groups.

*Mortality*: No studies reported on mortality.

*Asthma control*: Low-quality evidence from two studies (*n* = 1005) showed no differences between groups (MD 0.14, 95% CI −0.03 to 0.31) using the Paediatric Asthma Quality of Life Questionnaire.

*Adherence to asthma policies*: Evidence from two studies, graded low quality reported more schools that had received staff asthma training, had written asthma policies compared with control schools (OR 4.45, 95% CI 1.38–14.30).

*Asthma attacks*: Low-quality evidence from one study (*n* = 29 schools) reported that more intervention schools showed improvement in prevention or managing exercise-induced asthma attacks (OR 9.33, 95% CI 1.65–52.68) and more of these schools reported that staff administered salbutamol (OR 20.22, 95% CI 3.45–118.65).

*Absenteeism-related asthma*: There were no significant differences in the two groups, but skewed data made it difficult to interpret results (one study, *n* = 472).

*Staff preparedness – asthma knowledge*: Low-grade evidence from three studies (*n* = 640) showed a significant increase in the mean NAKQ score* in the intervention group compared with the control group (SMD 0.74, 95% CI 0.33–1.16).

*Newcastle Asthma Knowledge Questionnaire (two studies); Asthma General Knowledge Questionnaire (one study).

*Qualitative*: Key characteristics of successful interventions could not be identified as information on educational content and teaching attributes was not adequately reported.

## Implications for practice

Low-quality evidence suggests that asthma education improves knowledge and preparedness, but its benefit on the health and well-being of children with asthma in schools is unclear. Characteristics of successful interventions or resources required for their implementation cannot be determined from these studies.

## Implications for research

Future research should focus on high-quality trials of effectiveness and cost-effectiveness and report long-term clinical outcomes including exacerbations and mortality. Studies need to examine intervention content, modes of delivery, and the resources required for successful implementation.
